# Granulocyte-colony stimulating factor-associated aortitis in a woman with advanced breast cancer: a case report and review of the literature

**DOI:** 10.1186/s12885-019-6403-9

**Published:** 2019-12-16

**Authors:** Hideko Hoshina, Hiroyuki Takei

**Affiliations:** 1 Department of Breast Surgery and Oncology, Nippon Medical School, 1-1-5 Sendagi, Tokyo, Bunkyo 113-8603 Japan; 2 Department of Breast Surgery, Kikuna Memorial Hospital, Kikuna 4-4-27 Kouhoku, Yokohama, Kanagawa 222-0011 Japan

**Keywords:** Granulocyte-Colony stimulating factor (G-CSF), Aortitis, Breast Cancer, Pegfilgrastim, Filgrastim

## Abstract

**Background:**

Granulocyte-colony stimulating factor (G-CSF) is increasingly been used to prevent febrile neutropenia (FN) associated with the administration of chemotherapy for various cancers. The most common adverse effects of G-CSF are bone pain and injection-site reactions and aortitis has rarely been reported. We report herein a rare case of G-CSF associated with aortitis in a woman with advanced breast cancer.

**Case presentation:**

A 72-year-old woman with estrogen receptor-negative human epidermal growth factor 2-positive breast cancer with distant metastases in the lung was admitted. Her treatment was initiated with docetaxel in combination with trastuzumab and pertuzumab followed by the supportive use of a long-acting G-CSF, pegfilgrastim. After administration of pegfilgrastim on day 5, the patient had an intermittent fever (body temperature up to 39.6 °C) on day 9 which continued irrespective of taking levofloxacin. She visited our outpatient clinic on day 13 with no objective symptoms other than fever. Laboratory tests revealed a high neutrophil count (15,000/μl) and a high C-reactive protein (CRP) level (46.35 mg/dl) without any other abnormalities. There was no response upon administration of antimicrobial agents. An 18F-fluorodeoxyglucose-positron emission tomography/computed tomography (FDG-PET/CT) revealed thickening of the wall of the descending thoracic aorta and left pleural effusion. Therefore, thoracic aortitis induced by pegfilgrastim was suspected. On day 19, the fever resolved spontaneously followed by a gradual reduction in the neutrophil count and CRP level. In the follow-up CT, the aortic wall thickness and pleural effusion had disappeared.

**Conclusions:**

G-CSF may cause aortitis due to stimulation of the production of inflammatory cytokines. In case of high continuous fever after administration of pegfilgrastim, aortitis should be suspected unless there are other infectious findings.

## Background

In 2014, a long-acting granulocyte-colony stimulating factor (G-CSF) was approved for breast cancer by the national health insurance in Japan after which it has increasingly been administered to prevent febrile neutropenia (FN) without hospitalization. The most common adverse effects of G-CSF include bone pain and injection-site reactions [1]. G-CSF itself has no negative effects on cancer treatment. Furthermore, it has a favorable effect on maintaining a high relative dose intensity to cure the disease. On the other hand, according to the Japanese Adverse Drug Event Report (JADER) provided by the Pharmaceuticals and Medical Devices Agency (PMDA), aortitis is considered as one of the adverse effects of G-CSF although it has rarely been reported. Here, we report a case of aortitis induced by long-acting G-CSF administration to prevent FN in a woman with advanced breast cancer.

## Case presentation

**Fig. 1:**
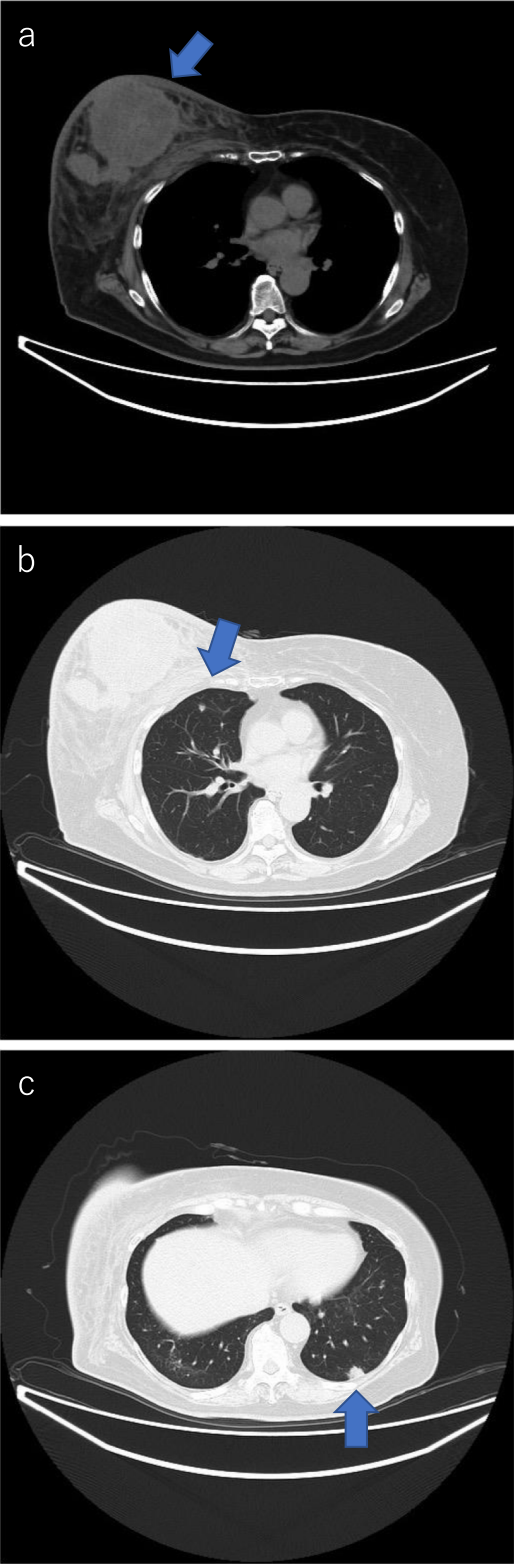
CT before chemotherapy shows left breast tumor, normal aorta (**a**), and multiple lung metastases (**b**, **c**)

A 72-year-old-woman with breast cancer who had already initiated treatment with chemotherapy (first cycle) visited our outpatient clinic with a chief complaint of high fever. She had no previous illness and no particular family history. On clinical examination, she was diagnosed with a clinical stage IV (T4d N2a M1) right breast cancer. A core needle biopsy revealed estrogen receptor-negative and human epidermal receptor 2-positive invasive ductal carcinoma of the right breast accompanied with lymph nodes metastases in the ipsilateral axilla. Computed tomography revealed distant metastases in the lungs (Fig. 1).

A chemotherapy regimen consisting of docetaxel 75 mg/m^2^, trastuzumab 8 mg/m^2^, and pertuzumab 840 mg was administered with dexamethasone 16.5 mg on day 1. Dexamethasone 16 mg was orally administered on days 2 to 4. As per the current guidelines, G-CSF administration is not recommended with the docetaxel regimen. However, G-CSF administration was chosen to ensure safer management of the elderly female patient with advanced-stage breast cancer. Therefore, pegfilgrastim, a long-acting G-CSF was subcutaneously administered on day 5. The patient complained of a high fever (body temperature up to 39.6 °C) in the morning on day 9 (day 5 of pegfilgrastim administration). Since then, the intermittent high fever persisted in the morning despite administration of levofloxacin which was prescribed for FN. The patient came to our outpatient clinic on day 13 (day 9 of pegfilgrastim administration) with high fever without any other subjective symptoms.

**Fig. 2:**
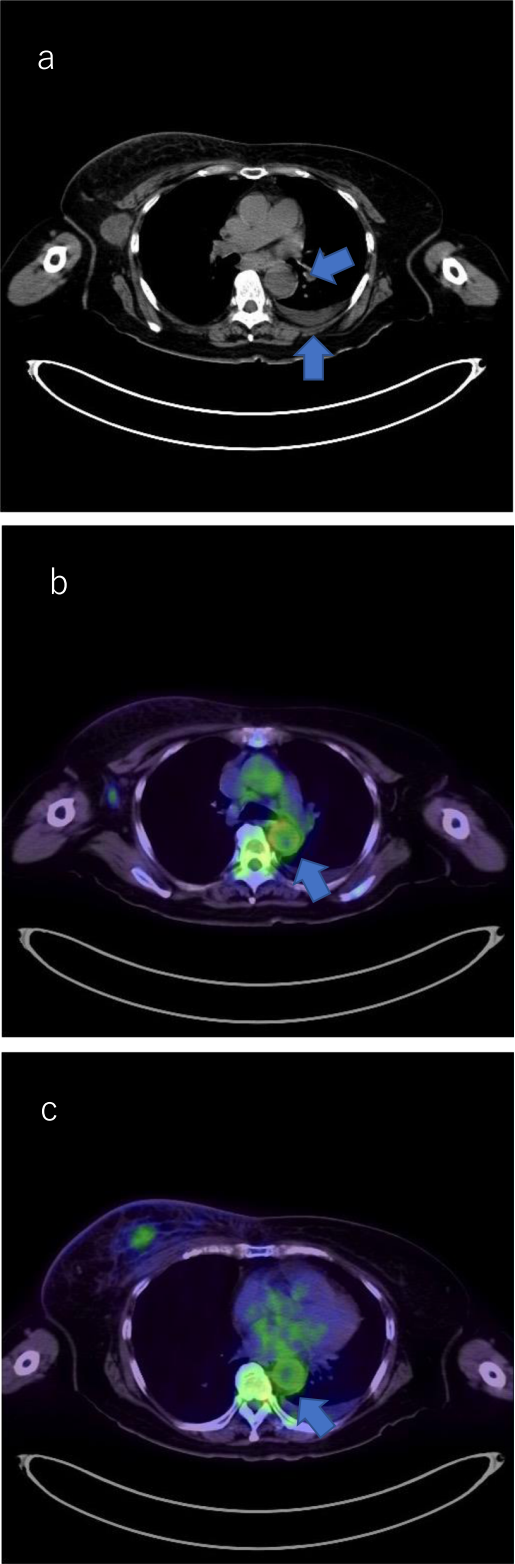
FDG-PET/CT shows the thickened wall of the thoracic aorta and left pleural effusion (**a**) with abnormal uptake of FDG (**b**, **c**)

The patient was conscious, physically well, and showed no infectious manifestations. Laboratory tests revealed a high neutrophil count (15,000/μl) and a high C-reactive protein (CRP) level (46.35 mg/dl) without any other abnormalities. Influenza antigen test was negative, and urinalysis was clear. Anti-nuclear antibody (ANA), myeloperoxidase-anti-neutrophil cytoplasmic antibody (MPO-ANCA), and serine proteinase3-anti-neutrophil cytoplasmic antibody (PR3-ANCA) were found to be negative later. However, interleukin-6 was slightly elevated (25.6 pg/ml). She continued to receive antibiotics (cefcapene pivoxil hydrochloride hydrate) because of suspected suffering an infectious disease although blood culture was negative. On day 15, 18F-fluorodeoxyglucose-positron emission tomography/computed tomography (FDG-PET/CT) was initially planned for identification of distant metastasis. However, chemotherapy was undertaken before FDG-PET/CT because a delay of initiation of chemotherapy was deemed inappropriate. FDG-PET/CT was performed at an initially reserved date in order to evaluate the presence of distant metastases except lung metastases. It revealed thickened wall of the descending thoracic aorta with an abnormal FDG uptake accompanied by left pleural effusion (Fig. 2). However, lung metastases disappeared, and we diagnosed the case as G-CSF-associated aortitis by the FDG-PET/CT.

On day 19, the fever reduced spontaneously. On day 21, the neutrophil count and CRP level reduced to 4940/μl and 13.29 mg/dl, respectively. The second cycle of chemotherapy was initiated with 30% reduced dose of docetaxel administered to the patient without pegfilgrastim. In the absence of pegfilgrastim administration, the docetaxel was reduced to 30% to ensure a safer management. The follow-up CT revealed the disappearance of both aortic wall thickness and pleural effusion. On day 1 of the third cycle of chemotherapy, the neutrophil count and CRP level were almost within the normal limits (4900/μl and 1.87 mg/dl, respectively). She has been continuing chemotherapy without any further complain of fever.

## Discussion and conclusion

In Japan, G-CSF-associated aortitis is very rare and occurs in just 0.47% of all cases of G-CSF administration based on the data from JADER [2]. In patients with cancer, aortitis occurs more frequently during chemotherapy with concomitant G-CSF compared to chemotherapy without G-CSF. The incidence of aortitis does not correlate with the type or regimen of chemotherapy. It has been reported more frequently in males than in females. In the United States of America, G-CSF-associated aortitis has been confirmed only in 15 cases as reported by the Adverse Event Reporting System (AERS) of the Food and Drug Administration (FDA) [3]. In these 15 cases, there was no correlation with the type of chemotherapy or gender. Except for five patients, all others recovered spontaneously.

G-CSF facilitates the differentiation and growth of neutrophils. However, it also stimulates the production of inflammatory cytokines [4] which may cause arteriosclerosis [5], aneurysm [6], and arteritis [7, 8]. Aortitis is classified into non-infectious and infectious and most of the non-infectious aortitis is caused by autoimmune disease relative to inflammatory cytokines [9]. In the present case, we excluded autoimmune disease because all of ANA, MPO-ANCA, and PR3-ANCA were within normal levels. We searched previously reported articles including abstracts by using the keywords “G-CSF” and “aortitis” in PubMed and CiNii (Citation Information by National Institute of Informatics). We also checked the references cited in the original articles, and finally identified 10 cases of G-CSF-associated aortitis including the present case (Table 1). The primary diseases included four breast cancer cases [10, 11], two lung cancer cases [12, 13], and one case of ovarian cancer [14]. G-CSF was used to prevent chemotherapy-induced FN in these cases. Additionally, there were two bone marrow donors [15, 16], and one case of drug-induced agranulocytosis. The latter case was induced by trimethoprim/sulfamethoxazole which was administered for aortitis syndrome [17].

**Table 1:** Reported cases of G-CSF-associated aortitis including the present case

References	[10]	[11]		[12]	[13]	[14]	[15]	[16]	[17]	Present case
Year	2018	2019		2009	2017	2018	2004	2016	2009	2019
Age	61	60	70	54	67	47	55	52	28	72
Gender	Female	Female	Female	Male	Female	Female	Female	Male	Male	Female
Primary disease	Breast cancer, Stage IV	Breast cancer	Breast cancer	Lung cancer, Stage III	Lung cancer, advanced	Ovarian cancer, Stage IIIC	Bone marrow donor	Bone marrow donor	Aortitis syndrome	Breast cancer, stage IV
G-CSF	PFG	G-CSF^a^	Filgrastim	Filgrastim	PFG	G-CSF^a^	Filgrastim	Filgrastim	G-CSF^a^	PFG
Period^b^	7 - 17	5 - 15	15 - 25	5 - 11	7 - 13	14 – 30^c^	2-14	6months	5-	4-14
Location of aortitis	Thoracic	Thoraco-abdominal		Abdominal	Thoracic, issection	Thoracic	Thoraco-abdominal	Abdominal and iliac aneurysms	Thoraco-abdominal and carotid arteritis	Thoracic
Diagnosis modality	CT, US	CT	PET/CT	CT, MRI	CT, US	CT	CT, MRI, US	CT	CT	PET/CT
Steroid treatment	None	PSL 60mg	PSL 60mg	None	mPSL 80mg	PSL 30mg	Corticosteroid1g	PSL 40mg	mPSL 1g	None

*G-CSF* granulocyte-colony stimulating factor, *mPSL* methylprednisolone, *PFG* pegfilgrastim, *PSL* prednisolone

^a^Details unknown

^b^Days with symptoms from G-CSF administration

^c^Days from chemotherapy

All cases were reported after 2004 suggesting that this disease is recently been recognized. All cases showed good performance status even with high fever and high CRP levels. In all the cases, aortitis was diagnosed by CT scan, FDG-PET/CT, magnetic resonance imaging (MRI), or ultrasound. In seven cases including the present case, high fever was noticed within 7 days of G-CSF administration. There were two cases of different arterial diseases other than aortitis (one case of iliac artery aneurysm and one case of dissection of descending aorta). It is unclear whether these arterial disorders correlated with G-CSF administration. Seven cases were treated with steroids (30–80 mg/day of oral prednisolone or 1 g/day of methylprednisolone). However, the high fever persisted for 7–17 days despite the use of steroids. On the other hand, the high fever persisted for 7–11 days without administration of steroids. There was no difference in the time to remission of aortitis with or without the use of steroids.

Interestingly, the five cancer cases where G-CSF was administered to prevent FN were advanced cancers. This signifies that inflammatory cytokines might be produced in larger quantities in advanced-stage cancer than in early-stage cancer. Accordingly, aortitis in patients with advanced-stage cancer should be considered as one of the differential diagnoses if there are long-lasting high fever and high CRP level after administration of G-CSF to prevent FN unless there are significant infectious manifestations.

## Data Availability

Not applicable

## Abbreviations

AERS: Adverse Event Reporting System
ANA: Anti-nuclear antibody
CiNii: Citation Information by National Institute of Informatics
CRP: C-reactive protein
FDA: Food and Drug Administration
FDG-PET/CT: 18F-fluorodeoxyglucose-positron emission tomography/computed tomography
FN: Febrile neutropenia
G-CSF: Granulocyte-colony stimulating factor
JADER: The Japanese Adverse Drug Event Report
MPO-ANCA: Myeloperoxidase-anti-neutrophil cytoplasmic antibody
MRI: Magnetic resonance imaging
PR3-ANCA: Serine proteinase3-anti-neutrophil cytoplasmic antibody
